# Bayesian Estimation of the True Prevalence and of the Diagnostic Test Sensitivity and Specificity of Enteropathogenic *Yersinia* in Finnish Pig Serum Samples

**DOI:** 10.1155/2015/931542

**Published:** 2015-10-11

**Authors:** M. J. Vilar, J. Ranta, S. Virtanen, H. Korkeala

**Affiliations:** ^1^Department of Food Hygiene and Environmental Health, Faculty of Veterinary Medicine, University of Helsinki, P.O. Box 66, Agnes Sjöbergin katu 2, 00014 Helsinki, Finland; ^2^Research and Laboratory Department, Risk Assessment Research Unit, EVIRA Finnish Food Safety Authority, Mustialankatu 3, 00790 Helsinki, Finland

## Abstract

Bayesian analysis was used to estimate the pig's and herd's true prevalence of enteropathogenic *Yersinia* in serum samples collected from Finnish pig farms. The sensitivity and specificity of the diagnostic test were also estimated for the commercially available ELISA which is used for antibody detection against enteropathogenic *Yersinia*. The Bayesian analysis was performed in two steps; the first step estimated the prior true prevalence of enteropathogenic *Yersinia* with data obtained from a systematic review of the literature. In the second step, data of the apparent prevalence (cross-sectional study data), prior true prevalence (first step), and estimated sensitivity and specificity of the diagnostic methods were used for building the Bayesian model. The true prevalence of *Yersinia* in slaughter-age pigs was 67.5% (95% PI 63.2–70.9). The true prevalence of *Yersinia* in sows was 74.0% (95% PI 57.3–82.4). The estimates of sensitivity and specificity values of the ELISA were 79.5% and 96.9%.

## 1. Introduction

Yersiniosis is a foodborne disease in humans, which is caused by* Yersinia enterocolitica* and to a lesser extent by* Yersinia pseudotuberculosis*, and it is the third most reported zoonotic disease in the EU [1].* Y. enterocolitica* infections have been associated with the consumption of pork products [2–4]. Often healthy pigs are asymptomatic carriers of* Y. enterocolitica*; and they are a major reservoir for human pathogenic strains [3, 5, 6].

Diagnostic tests are used for prevalence surveys. Ideally, true prevalence should be estimated from apparent prevalence adjusting for the diagnostic test sensitivity and specificity [7]. It is a common observation that the sensitivity and specificity estimates differ among validation studies, which can be explained due to differences among reference population and sampling strategies [8]. Differences in sensitivity and specificity between diagnostic methods can result in a considerable variation in prevalence estimations, when they are not taken into account. For this reason, reliable estimates of sensitivity and specificity of diagnostic tests are necessary.

Various methods have been described for detection of antibodies against enteropathogenic* Yersinia* in serum samples of pigs at farms and in juice extracted from tonsils and meat at farms and slaughterhouses [9–14]. However, these diagnostic tests have different sensitivities and specificities making the direct comparison of the results difficult.

The true prevalence can be estimated from an apparent prevalence by using frequentist or Bayesian methods. For example, frequentist methods assume that true prevalence is a fixed unknown quantity by which a randomly chosen individual from the population is infected [7]. One of the estimators of true prevalence is the Rogan-Gladen estimator [15]. The Bayesian inferences have been advocated as more flexible and useful to solve complex problems [16], and they allow the incorporation of prior information in addition to the data. The Bayesian approach has been used in validation of diagnostic methods, providing a reliable estimate of the sensitivity and specificity when there is more than one diagnostic test but no gold standard. An example of this is the evaluation of the diagnostic test for detection of classical swine fever [17]. Also, a Bayesian hidden variable model has been developed to study the occurrence of foodborne pathogens in the pork production chain [18].

The true prevalence of* Y. enterocolitica* in pigs sampled in farms and slaughterhouses is not directly noticeable. These should be estimated using the information from the apparent prevalence and the sensitivity and the specificity of the diagnostic test [7]. Neither the sensitivity nor the specificity of the commonly used tests is known with certainty, which introduces additional uncertainty when adjusting apparent prevalence. Using a Bayesian analysis, the true prevalence of enteropathogenic* Yersinia* in serum of Finnish pigs has been estimated. The sensitivity and specificity of the diagnostic test were also estimated.

## 2. Materials and Methods

### 2.1. Definitions

Definitions of prevalence, sensitivity, and specificity were considered as defined by Greiner and Gardner [8] and Thrusfield [19]. Apparent prevalence (Ap) is the proportion of the pig population that tests positive using a diagnostic method, and true prevalence (Tp) is the proportion of truly infected pigs in that population. The sensitivity (Se) of a diagnostic test is the proportion of infected animals that the test detects as positive. Specificity (Sp) of a diagnostic test is the proportion of noninfected animals that the test detects as negative.

### 2.2. Modelling Approach

The model was built in two steps using the Bayesian analysis to calculate the posterior probabilities, depending on data and prior distribution. The model estimated the true prevalence of* Yersinia* in serum samples. The prior distribution of the true prevalence was estimated based on a systematic review in the first step of the model, and later on introduced in the second step.

#### 2.2.1. First Step

The first step is a model to estimate the prior distribution of the true prevalence and to estimate the prior distribution for sensitivity and specificity of ELISA test.

*Systematic Review*. The objective of the systematic review was to assess the apparent prevalence of* Yersinia* in serum samples in slaughter-age pigs and sows from farms in Finland. For this review, the questions, type of intervention, population, and outcome were used to create the inclusion criteria [20]: any study or survey that evaluates the presence of and risk factors for antibodies against enteropathogenic* Yersinia* in serum samples from slaughter-age pigs and sows in farms using a commercially available enzyme-linked immunosorbent assay (ELISA) kit (Pigtype Yopscreen, Labor Diagnostik, Leipzig, Germany).

Papers written in any language were searched, and when data was published in different articles by the same authors or in reviews, we considered them only once to avoid duplication. Data from unpublished studies was not available. The keywords used for the search were* Yersinia*, pigs or pig farms, and prevalence or seroprevalence as words in the titles or the abstracts when searching in the National Center for Biotechnology Information (NCBI) PubMed database or as the topic when searching in Web of Science. We also looked over the reference lists of the relevant papers and in auxiliary data sources, such as the Google search engine.

All studies identified were assessed against the defined inclusion criteria. Selection of studies was carried out in two stages: the first stage by screening the title and abstract of the manuscripts and the second stage by screening the full text. The number of publications selected from the systematic review was 4, while 8 manuscripts were excluded from the review because they failed in at least one of the inclusion criteria (the list of manuscripts is shown in Table 1); for example, the diagnostic tests described in the manuscripts were different from the commercial ELISA kit, and thus the sensitivities and specificities, or the samples were taken at the slaughterhouses.

The data collected from each of the manuscripts was as follows: the number of positive pigs and the number of positive farms (or herds), the number of sampled pigs and the number of sampled farms (or herds), age of sampled pigs, methodology used for analysing the samples, when and where (country level) the study was carried out, the authorship, and the published journal. We took into account data taken from tables when there was any inconsistency between data of the text and the tables. Data was collected from the selected studies and recorded in Excel (Microsoft Corp., Redmond, WA).

*Construction of the Model with Literature Data*. Information on number of positive pigs (or herds) and number of sampled pigs (or herds) obtained from the systematic review was used as observed data. Noninformative (uniform) prior distributions Beta(1,1) were assigned as the prior distributions of pig and herd level true prevalence in the literature data, since it is commonly used as prior distribution for binomial proportions [29] when the prevalence is a random variable. As result, the posterior distributions of the prevalence, based on literary data, were used as informative prior distributions in the second stage below. In this way, the information from previous literature becomes utilized, with the assumption that the selected collection of literature represents roughly similar prevalence in pig populations in Finnish studies.

Information provided by the validation report published by the manufacturer of ELISA test (Pigtype Yopscreen, Labor Diagnostik, Leipzig, Germany) was used to estimate the prior distributions for sensitivity and specificity of the serological analyses. The sensitivity of ELISA was modelled using the validation report of the manufacturer, where *x* out of *n* infected animals tested positive; then beta(*x* + 1, *n* − *x* + 1) gives the posterior distribution of sensitivity, assuming a binomial model and uniform prior distribution for sensitivity [30].

Estimates of the pig and herd prevalence were calculated in this first step by using a model mathematically similar to the one use in the second step. The obtained posterior medians and 95% PI (probability interval) of the prevalence were used as inputs in the software Betabuster (downloaded from http://www.epi.ucdavis.edu/diagnostictests/betabuster.html) to obtain the shape parameters for the prior beta distributions to be introduced in the second step of the modelling. When the estimated value was between 0 and 0.5, the 95th percentile was chosen, and when the estimated value was between 0.5 and 1 the 5th percentile was chosen, according to the instructions provided by the copyright holders of Betabuster. The beta prior distribution of the specificity was also obtained using this procedure.

#### 2.2.2. Second Step

The second step is a model to estimate the pig and herd true prevalence of* Yersinia* in serum samples in Finland.

*Collection and Analyses of Samples*. The study was carried out in Varsinais-Suomi region that accounts for 28% of the total pigs in Finland (pig census from Matilda, Agricultural Statistics of Ministry of Agriculture and Forestry, 2010). The number of pigs to be sampled was calculated as previously described by Vilar et al. (2013) [21]. Individual serum samples from 120 slaughter-age pigs (50 kg or more) and 107 sows were collected in 16 farms and analysed for occurrence of antibodies against* Yersinia*. The total number of sampled pigs and the number of pigs positive using the diagnostic test in each farm were recorded to calculate the prevalence at pig and herd level.

Serum samples were tested for the presence of* Yersinia* antibodies by using a commercially available ELISA kit (Pigtype Yopscreen, Labor Diagnostik, Leipzig, Germany), with a cut-off optical density (OD) value of 0.2 according to the manufacturer's instructions.

**Figure 1:**
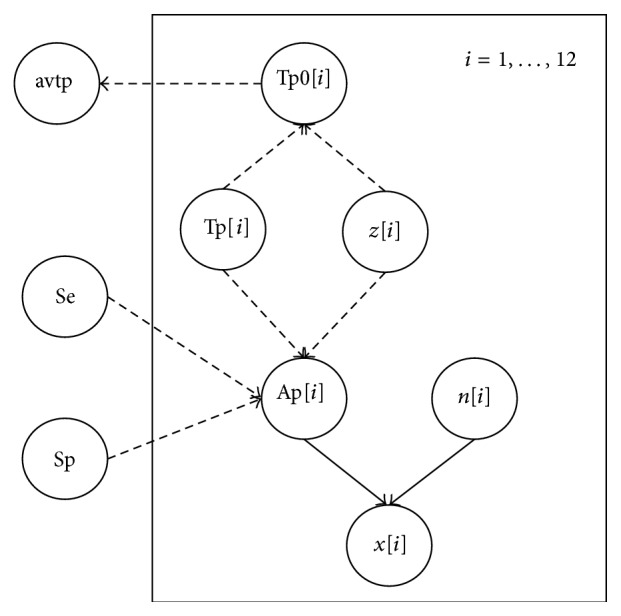
Graphical model used for the Bayesian analyses to estimate the true prevalence, presenting the conditional dependency structure between variables. The observed variable, *x*[*i*], is the number of pigs detected positive (Ap) in the sample of size *n*[*i*]. The priors are beta distributions for the sensitivity (Se) and specificity (Sp) of the diagnostic test. Tp[*i*] is the true prevalence, for a truly positive farm. Since a farm can be truly nonpositive, the actual true prevalence is Tp0[*i*] = Tp[*i*]∗*z*[*i*], where *z*[*i*] represents an indicator variable that a farm is truly positive.

*Construction of the Model with Observed Data*. A binomial sampling model was assumed as the population size in each farm was large enough compared with the sample size. The size of the farms was on average 630 slaughter-age pigs and 306 sows. The average number of slaughter-age pigs sampled in each farm was 13, and the average number of sows sampled in each farm was 9. The Bayesian model to estimate true prevalence was mathematically constructed from the conditional distributions (shown in Figure 1):

  
*x*[*i*]∣Ap[*i*], *n*[*i*]; ~Bin(Ap[*i*], *n*[*i*]),
  Ap[*i*] ← Tp[*i*]∗*z*[*i*]∗Se + (1 − Tp[*i*]∗*z*[*i*])∗(1 − Sp),
  Tp[*i*] ~ beta(*α*Tp, *β*Tp),
  Se ~ beta(*α*Se, *β*Se), Sp ~ beta(*α*Sp, *β*Sp),
  
*z*[*i*] ~ dbern(tau), tau ~ beta(*α*tau, *β*tau),
  Tp0[*i*] ← Tp[*i*]∗*z*[*i*],

where *x*[*i*] is the observed number of pigs that tested positive in farm *i*, and Ap[*i*] is the probability that a randomly selected pig from farm *i* tests positive, and *n*[*i*] is the sample at that farm. Ap[*i*] is the apparent prevalence in each farm. Tp[*i*] is the true prevalence for a truly positive farm, that is, prevalence when there is at least one truly positive animal. Since a farm can be truly nonpositive, the actual true prevalence is Tp0[*i*] = Tp[*i*]∗*z*[*i*], where *z*[*i*] represents an indicator variable that a farm is truly positive; that is, at least one animal would be truly positive. The actual true prevalence Tp0[*i*] for a farm is effectively described as a zero inflated distribution with a point probability mass at zero. The *z* variable is also needed for the correct interpretation of Tp as the true prevalence for pigs in infected farms, because also the prior of Tp is based on previous data providing posterior distribution of true prevalence in infected herds.

There is variation between farms in true prevalence so that it is not realistic to assume a common prevalence for all farms. These differences are accounted by modelling prevalence as a farm specific parameter. Finally, tau represents herd true prevalence, the proportion of truly positive herds. The prior of tau was also calculated based on the literary review. The independent beta prior distributions obtained in the first step of this paper were used to take into account the uncertainty in the prevalence as well as in the diagnostic test sensitivity and specificity [31]. Thus, the priors for prevalence were based on the literature data, expressed as beta distributions, beta(*α*Tp, *β*Tp), conditionally based on that the population is infected.

Bayesian analysis was also used for upscaling estimates for a larger finite population, assuming that it is similar to the study population. Data of pig census was obtained from Matilda (Agricultural Statistics of Ministry of Agriculture and Forestry, 2010) and used to calculate the apparent and true prevalence of* Yersinia* in the whole of Finland. The upscaling was based on evaluating the average of actual true prevalence in the study farms avtp = mean(Tp0[1,…, 12]), which represents the actual true prevalence in the study population of pig herds. Assuming that these are representative of all herds in the census, the expected number of positive pigs is avtp times the census size. Posterior distribution of this was computed.

Models were constructed in WinBUGS 1.4.3, and the graphical representation is shown in Figure 1. Inferences were based on 50000 iterations after a burn-in for convergence of 1000 iterations. Results of the posterior probability distributions are summarized by the median and the probability intervals (PI).

### 2.3. Sensitivity Analysis

Different prior distributions and noninformative prior distributions were used to perform the sensitivity analysis. Different prior distributions for the prevalence were used in the set of priors 1. Noninformative prior distributions for prevalence and sensitivity were introduced in the set of priors 2. Later on, the posterior median values obtained were compared for significant differences by a general linear model for repeated measures.

## 3. Results and Discussion

In this study a Bayesian analysis was used to provide reliable information on the prevalence of enteropathogenic* Yersinia* in pigs sampled at farms in Finland, and also to provide useful and relevant information of the diagnostic test commonly used for their detection.  Table 2 shows the estimates of the posterior distributions of the pig and herd true prevalence from the model built in the first step. The sensitivity and the specificity of the diagnostic tests are also shown. These values were used when building the model of the second step. The prior distribution of the sensitivity of the commercial ELISA used to test the serum samples for the presence of* Yersinia* antibodies was beta(63,29), and the specificity prior distribution was Sp ~ beta(6.0,1.1).

The results of the posterior probabilities obtained in the second step are shown in Table 3. The posterior probability of the true prevalence of enteropathogenic* Yersinia* in slaughter-age pigs had a median value of 67.5%. The predicted total number of* Yersinia* positive slaughter-age pigs was 329,000 (308,400–345,800) out of 487776 slaughter-age pigs in the whole of Finland. The posterior probability of the true prevalence of enteropathogenic* Yersinia* in sows had a median value of 74.0%. The true prevalence of enteropathogenic* Yersinia* in serum samples from slaughter-age pigs estimated in the present study was lower than apparent prevalence reported previously [14, 21, 32–38]. However, those studies were based on a frequentist approach. On the other hand, when there is no prior information, frequentist analysis produces good estimates of prevalence [39], and this would correspond to Bayesian analysis with noninformative priors. However, some background information about sensitivity and specificity is needed in both cases.

Table 3 also presents the sensitivity analysis conducted by comparing the model with the original set of priors with the other sets of priors. Sensitivity analysis serves to illustrate how prior knowledge could affect the posterior estimates [40]. Although the values were not exactly similar, no significant differences were found between posterior medians and their PI. The model used was not very sensitive to the choice of priors, as the posterior probabilities for the three sets of priors were similar across the pig populations.

It has been reported that prevalence is associated with the prevalence of* Yersinia* in tonsils [10] and in faeces [11, 21]. However, the prevalence values of* Yersinia* are usually higher than the prevalence values of* Y. enterocolitica* in faeces, both collected in farms [21]. This difference can be explained because the antibodies are usually present long after an infection starts [10, 41] and because the commercial ELISA test used in the present study detects antibodies based on the outer membrane proteins and thus detects infections with all pathogenic* Yersinia*. However, in Finland the prevalence of* Yersinia pseudotuberculosis* has been reported to be less than 8% [42, 43].

Sensitivity and specificity of the ELISA diagnostic test were 79.5% and 96.9%, respectively. The estimations obtained indicated that the commercial ELISA test, although good, had lower sensitivity and specificity than that previously reported by the manufacturer. Some studies [11–13] have used the commercial ELISA test but the accuracy characteristics of 100% sensitivity and 100% specificity reported by the manufacturer have not been discussed. Furthermore, no tests can be considered as having both 100% sensitivity and 100% specificity, as it is thought that estimates vary among validation studies, such as sampling strategies, technical variation between laboratories, choice of gold standard, and state of infection [8].

## 4. Conclusions

By using the estimates obtained by the Bayesian analysis it was possible to estimate the true prevalence of* Yersinia* in the population under study, without sampling all animals. Consequently, the model constructed in the present study can be extended when studying a country's population, which would overcome the logistic difficulties of sampling high numbers of animals. The Bayesian approach provided a reliable estimate of the sensitivity and the specificity of the commonly used commercial ELISA for detection of enteropathogenic* Yersinia*.

## Figures and Tables

**Table 1:** List of papers selected from the systematic review to obtain the prior estimates of pig and herd level prevalence of enteropathogenic *Yersinia* in Finland and the list of papers from the systematic review that were excluded.

Author	Reference	Sample (location)	Country	Number of positive pigs/number of sampled pigs (%)
Included				
Vilar et al. [21]	Foodborne Pathog. Dis., 2013, 10: 595–602	Serum and faeces (farm)	Finland	182/334
Virtanen et al. [14]	Appl. Environ. Microbiol., 2012, 78: 3000–3003	Serum and faeces (farm)	Finland	31/65
Von Altrock et al. [11]	Berl. Munch. Tierarztl. Wochenschr., 2006, 119: 391–396	Serum and faeces (farm)	Germany	573/900
Von Altrock et al. [13]	Foodborne Pathog. Dis., 2011, 8: 1249–1255	Serum (farm and slaughter)	Germany	574/900
Not included				
Vanantwerpen et al. [9]	Prev. Vet. Med., 2014, 116: 193–196	Meat juice (slaughter)	Germany	4652/7047
Meemken et al. [22]	Prev. Vet. Med., 2014, 113: 589–598	Meat juice (slaughter)	Germany	1805/3323
Stojek et al. [23]	Bull. Vet. Instit. Pulawy., 2010, 54: 309–313	Serum (farm)	Poland	39/226
Nesbakken et al. [24]	Emerg. Infect. Dis., 2007, 13: 1860–1864	Serum and faeces (farm)	Norway	27/1073
Nesbakken et al. [25]	Int. J. Food Microbiol., 2006, 111: 99–104	Serum and faeces (farm)	Norway	163/239
Nesbakken et al. [26]	Int. J. Food Microbiol., 2003, 80: 231–240	Serum (slaughter)	Norway	21/24
Thibodeau et al. [27]	Vet. Microbiol., 2001, 82: 249–259	Serum and faeces (slaughter)	Canada	192/291
Skjerve et al. [28]	Int. J. Food Microbiol., 1998, 45: 195–203	Serum (slaughter)	Norway	1774/4029

**Table 2:** Estimates of the posterior distributions of the prevalence of enteropathogenic *Yersinia* in serum at pig and herd level. Estimates were obtained based on a systematic review of the literature and used for building the model in the second step.

Sample	Parameter	Median (95% PI)	Beta distribution	
			Alpha	Beta
Serum	Herd prevalence	0.879 (0.418–0.994)	4.157	1.435
	Pig prevalence slaughter-age pigs	0.883 (0.694–0.992)	16.461	3.049
	Pig prevalence sows	0.901 (0.469–0.995)	4.779	1.415
	Sensitivity^a^		63	29
	Specificity^b^	1	6.024	1.051

PI: the 95% probability intervals.

^a^Information obtained from the validation report of the commercial ELISA test.

^b^Specificity value of 1 was replaced by a most likely value of 0.9.

**Table 3:** Probability posterior estimates for the prevalence of enteropathogenic *Yersinia* in serum at pig and herd level. Sensitivity and specificity for the diagnostic test are also shown. Estimates with different set of priors are also presented, as part of sensitivity analyses.

Sample	Parameter	Posterior estimates, median (95% probability interval)		
		Original model	Set of priors 1	Set of priors 2
Serum	Sensitivity^a^	0.795 (0.736–0.848)	0.802 (0.744–0.855)	0.919 (0.833–0.990)
	Specificity^a^	0.969 (0.853–0.999)	0.961 (0.826–0.998)	0.978 (0.894–0.999)
	Herd prevalence slaughter-age pigs	0.776 (0.522–0.937)	0.782 (0.528–0.943)	0.777 (0.522–0.936)
	Herd prevalence sows	0.868 (0.625–0.981)	0.868 (0.628–0.979)	0.869 (0.634–0.978)
	Pig prevalence slaughter-age pigs	0.675 (0.632–0.709)	0.645 (0.572–0.709)	0.654 (0.613–0.689)
	Pig prevalence sows	0.740 (0.573–0.824)	0.749 (0.586–0.829)	0.720 (0.578–0.803)

^a^Estimates calculated considering all pigs, that is, slaughtered-age pigs and sows.

Herd prevalence and pig prevalence are based on the estimates of tau and Tp, respectively.

The following are other priors for sensitivity analysis.

Set of priors 1: using different prior distributions for pig prevalence.

Set of priors 2: using noninformative prior distributions beta(1, 1) for pig prevalence and for sensitivity beta.
